# Mouse Transcobalamin Has Features Resembling both Human Transcobalamin and Haptocorrin

**DOI:** 10.1371/journal.pone.0020638

**Published:** 2011-05-31

**Authors:** Katrine Hygum, Dorte L. Lildballe, Eva H. Greibe, Anne L. Morkbak, Steen S. Poulsen, Boe S. Sorensen, Torben E. Petersen, Ebba Nexo

**Affiliations:** 1 Department of Clinical Biochemistry, Aarhus University Hospital, Aarhus, Denmark; 2 Institute of Biomedical Sciences, Panum Institute, University of Copenhagen, Copenhagen, Denmark; 3 Department of Molecular Biology, Aarhus University, Aarhus, Denmark; Bauer Research Foundation, United States of America

## Abstract

In humans, the cobalamin (Cbl) -binding protein transcobalamin (TC) transports Cbl from the intestine and into all the cells of the body, whereas the glycoprotein haptocorrin (HC), which is present in both blood and exocrine secretions, is able to bind also corrinoids other than Cbl. The aim of this study is to explore the expression of the Cbl-binding protein HC as well as TC in mice. BLAST analysis showed no homologous gene coding for HC in mice. Submaxillary glands and serum displayed one protein capable of binding Cbl. This Cbl-binding protein was purified from 300 submaxillary glands by affinity chromatography. Subsequent sequencing identified the protein as TC. Further characterization in terms of glycosylation status and binding specificity to the Cbl-analogue cobinamide revealed that mouse TC does not bind Concanavalin A sepharose (like human TC), but is capable of binding cobinamide (like human HC). Antibodies raised against mouse TC identified the protein in secretory cells of the submaxillary gland and in the ducts of the mammary gland, i.e. at locations where HC is also found in humans. Analysis of the TC-mRNA level showed a high TC transcript level in these glands and also in the kidney. By precipitation to insolubilised antibodies against mouse TC, we also showed that >97% of the Cbl-binding capacity and >98% of the Cbl were precipitated in serum. This indicates that TC is the only Cbl-binding protein in the mouse circulation. Our data show that TC but not HC is present in the mouse. Mouse TC is observed in tissues where humans express TC and/or HC. Mouse TC has features in common with both human TC and HC. Our results suggest that the Cbl-binding proteins present in the circulation and exocrine glands may vary amongst species.

## Introduction

In humans, two circulating cobalamin (Cbl) -binding proteins have been identified and characterized: transcobalamin (TC) and haptocorrin (HC). TC transports Cbl into all cells of the body via binding to CD320, a recently identified membrane receptor [1]. The function of HC remains unknown, but the protein carries the major part of circulating Cbl and it is present in secretions such as saliva and milk [2]. A characteristic feature of HC is that it has a higher affinity for a wider spectrum of Cbl forms than TC. Moreover, recent studies have shown that so-called Cbl-analogues capable of binding to HC are present in the human circulation [3].

TC and its encoding gene, TCN2, have been observed in all animals including mouse, rat, monkey, hog, horse, opossum, cow, dog, chicken, and chimpanzee [4]. HC and/or its encoding gene, TCN1, have been found in hog, cow, dog, rhesus monkey, horse, chimpanzee, platypus [5], and rabbit [6]. In hen, the Cbl-binding protein has been purified and partly characterized [7], but with no definitive conclusion as to its classification as TC or HC. In rat [8] and opossum [9], the presence of HC has been suggested by indirect methods, but without protein purification and final classification.

Since mice are commonly used experimental animals and serve as physiological models, we wanted to explore the expression of the Cbl-binding protein HC as well as TC in mice.

## Materials and Methods

### DNA/BLAST analysis

DNA/BLAST sequence search and analysis was performed using the National Center for Biotechnology Information (NCBI) website [10]. The nucleotide sequences for human TCN1 were compared with the mouse sequences by use of the Basic Local Alignment Search Tool (BLAST). The human TCN1 genomic sequence was compared to the mouse genome by use of the Map Viewer function. Likewise, the sequence of TCN2 was searched.

### Experimental animals and tissues

A wide variety of different inbred mouse strains were obtained from the animal section, Institute of Medical Microbiology and Immunology, Aarhus University, Denmark and from the Institute of Biomedical Sciences, Panum Institute, University of Copenhagen, Denmark. All mice were in care of the animal section enjoying standard living conditions for laboratory mice. They were part of no experimental studies while alive and were destined to be killed independently of the present study. On the day the mice were sacrificed, each mouse was taken directly from the cage and killed immediately by cervical dislocation. Tissues removed were instantly placed on dry ice or in liquid nitrogen and subsequently stored in aliquots at – 80 °C. All mammary glands were taken from lactating mice. Blood was either drawn from the orbital sinus (serum) or from the vena cava using a heparin-coated syringe (plasma); in both settings prior anesthesia of the mice was applied.

### Pre-treatment of tissues and blood for protein analysis

Keeping the sample on ice, 10 µL homogenisation buffer (10 mM PIPES pH 7.4 (Sigma, Brøndby, Denmark), 1 mM EGTA (Sigma, Brøndby, Denmark), 3 mM MgCl_2_, 6H_2_O (Merck, Damstadt, Germany), 400 mM NaCl, 2 tablets per 25 mL buffer of proteinase inhibitor cocktail (Cat. No. 11697498001, Roche Diagnostics, Mannheim, Germany)) were added per mg tissue. The tissue was homogenized using a tissue ruptor (Qiagen, Copenhagen, Denmark) and sonicated (MSE probe universal) 3 times 10 s. The sample was centrifuged for 40 min at 20000 G at 4°. The supernatant was kept at – 20 °C until analyzed. Mouse blood was stored at −20° until analyzed.

For comparative analysis, human TC was obtained from partly purified spermal plasma [11] and human HC was obtained either from a pool of saliva originally collected in relation to a study of the salivary content of HC [12] or from a patient with a high plasma concentration of HC [13].

### Unsaturated cobalamin-binding capacity and total cobalamin

Unsaturated Cbl-binding capacity (UB12BC) was measured by adding radiolabeled Cbl (Cbl*) ([^57^Co]cyanocobalamin, Kem-En-Tec, Taastrup, Denmark) with a known specific activity. Unbound Cbl was removed by addition of coated charcoal while employing a previously described method [14].

Samples with a Cbl-binding capacity above 0.9 nM were diluted five (mammary glands) or 100–1000 (parotid and submaxillary glands) times in 0.1% PBS (0.1 M phosphate buffer pH 8.0 (Ampliqon, Skovlunde, Denmark), 1 g/L bovine serum albumin (analyzed for Cbl content, Sigma)) prior to analysis.

The total Cbl concentration was measured using Cobas 6000 E immunoassay system (Roche Diagnostics, Hvidovre, Denmark) with a detection range of 55–1476 pM. Samples were diluted with diluents supplied by the manufacturer prior to analysis.

### Characterization of the unsaturated Cbl-binding protein

The protein's binding affinities for Cbl and cobinamide (Cbi) were explored in crude protein extracts of mouse submaxillary glands, and for human TC and HC. The analysis was performed in a competitive assay as described previously [15].

The glycosylation status of the Cbl-binding protein from extracts of mouse submaxillary glands was explored by precipitation by Concanavalin A sepharose (Con A Sepharose 4B, Amersham Biosciences, Uppsala, Sweden), a lectin known to bind α-D-mannopyranosyl, α -D-glucopyranosyl, and sterically related residues. For each sample (mouse submaxillary gland extract, human TC, and human plasma HC), the UB12BC was adjusted to 2 nM by dilution with binding buffer (20 mM Tris-HCl pH 7.4, 0.5 M NaCl) and 600 µL of each sample was incubated with 10 µL 5 nM Cbl*, approx. 6500 cpm, for 30 min at room temperature (RT). 100 µL Con A sepharose (prepared according to the manufacturer's instructions) and 200 µL binding buffer were added and the sample was incubated 1 h at RT before centrifugation for 5 min at 8.0 G. The Cbl* present in the supernatant and the precipitate was measured by gamma counting (1470 Wizard, Wallac Automatic Gamma Counter).

### Purification and sequencing of mouse submaxillary Cbl-binding protein

The unsaturated Cbl-binding protein present in 300 ml crude protein extract from more than 300 mouse submaxillary glands was purified to homogeneity by affinity chromatography where sepharose-coupled hydroxyl-Cbl was used, essentially as previously described [16].

8 mL sepharose slurry (EAH sepharose 4B GE Healthcare, Uppsala, Sweden) was washed with 200 mL 0.1 M NaH_2_PO_4_ pH 7.5. Thereafter, 10 mL of 1 mg/mL hydroxy-Cbl (GEA, Copenhagen, Denmark) mixed with 3 mL 0.1 M NaH_2_PO_4_ pH 7.5 was applied to the sepharose, gently mixed, and incubated for 5 hours at 57° while gently mixing the solution every 20-30 min. 30 µL 20% sodium azide was added per 15 mL B12-sepharose, and the sepharose was placed at 4 °C. Prior to use, the B12-sepharose was washed with 50 volumes of 0.1 M NaH_2_PO_4_ pH 7.5 followed by 30 volumes demineralised water and six volumes of 0.1 M NaH_2_PO_4_ pH 7.5. The mouse submaxillary extract was applied to the column with a flow rate adjusted to allow the extract to incubate with the B12-sepharose for ≥5 min. The column was washed with approx. 15 volumes of 0.1 M Tris pH 7.5 with 1 M NaCl and with approx. three volumes of 0.1 M NaH_2_PO_4_ pH 7.5. Absorbed Cbl-binding protein was eluted by incubating the column at 37 °C over night and by applying approx. two volumes of 0.1 M NaH_2_PO_4_ pH 7.5 preheated to 37 °C. The elution procedure was repeated and the pooled eluate was dialyzed employing 14 kDa cut-off dialysis tubes (Medicell International, London, U.K.) against 0.1 M NaH_2_PO_4_ pH 7.5 for 6 hours at 4 °C and against demineralised water at 4 °C over night, lyophilised (HETOVAC), and kept at −20 °C until further use.

Protein purity was evaluated by Coomassie-stained 12% SDS-PAGE by running 20 µL 275 mg/L protein on precast 12% Tris-HCl gel (Bio Rad, Hercules, California, USA).

Two major bands from an SDS-gel were cut out of the gel and after reduction with dithioerythritol following incubation with iodoacetamide, the samples were digested with trypsin, and extracted peptides were analyzed on a Voyager-DE MALDI-TOF mass spectrometer (Applied Biosystems, CA, USA). The data were searched using the Mascot search engine [17].

### Identification and alignment of protein sequences

Protein sequences of human HC (NP_001053), human TC (NP_000346), human IF (NP_005133), mouse TC (NP_056564), and rat TC (NP_071979) were found in the protein database of the NCBI website [18] using search term “TCN” and the respective organisms. Protein sequence alignment was done using clustalW2 at the homepage of the European Molecular Biology Laboratory- European Bioinformatics Institute (EMBL EBI) using default settings [19], [20].

### Antibodies against mouse TC: production, test, and use

Polyclonal antibodies (anti-mouse TC) were produced by immunizing a rabbit six times with approx. 7 µg purified protein mixed with Freund's incomplete adjuvant (Sigma, Brøndby, Denmark) to a volume of 600 µL. Immunizations were given subcutaneously at weeks 1, 3, 5, 7, 11, and 15. After 19 weeks, the rabbit was bled to death and serum was collected and stored in aliquots at −20° until the gamma globulin fraction was purified as described previously [21]. The purified, lysophilised anti-mouse TC was stored at −80 °C.

To test the binding specificity of anti-mouse TC, it was used in standard Western blot of 10% native PAGE (MiniProtean ® TGX TM, BioRad, Hercules, CA, USA) analysis of serum, crude extract of submaxillary gland, and purified mouse TC. Secondary antibody was polyclonal goat anti-rabbit immunoglobulins/HRP (DAKO, Glostrup, Denmark) and the results were visualised using UVP BiospectrumAC Imaging System (AH diagnostics).

Anti-mouse TC was used in a precipitation study: 1 mg purified gamma globulin of rabbit anti-mouse TC was coupled to proteinA/G+agarose according to the manufacturer's instructions (SantaCruz Biotech, California, USA). The binding capacity was calculated based on experiments where increasing amounts of anti-mouse TC-agarose were added to a constant submaxillary gland crude protein extract (UB12BC 25 nM) and by measuring the UB12BC before and after 1 h of incubation at RT while rolling. To evaluate the presence of other Cbl-binding proteins in mouse serum, the sum of UB12BC and Cbl was calculated and a 2-fold excess binding capacity of anti-mouse TC-agarose was added to the sample. UB12BC and Cbl were measured before and after absorption as described above and the fraction of precipitated UB12BC and Cbl was calculated.

For immunohistochemistry, submandibular glands and lactating mammary glands were taken from five mice and fixed in ice-cold 4% paraformaldehyde in 0.1 M phosphate buffer pH 7.4. The tissue samples were embedded in paraffin and cut into 10-mm sections using a microtome. The sections were incubated for 5 min in 2% bovine serum albumin followed by 18 h at 4°C with the primary antiserum diluted 1∶3200. For visualisation of the immunoreactions, the sections were incubated for 1 h with biotinylated porcine anti-rabbit immunoglobulins (code no. E 353,DakoCytomation), diluted 1∶40 as the second layer, followed by StreptABComplex/horseradish peroxidase (code no. E 353, DakoCytomation) diluted 1∶200 as the third layer, and, finally, stained by means of 3,3-diaminobenzidine for 30 min. The sections were counterstained with hematoxylin. As a control for non-specific staining, the primary antiserum was replaced by pre-immune serum from the same rabbit in the same dilutions.

### mRNA expression of TC in mouse tissues

Total RNA was purified from mouse tissues by the use of QIAamp RNA purification kit (Qiagen, Hilden, Germany) according to the manufacturer's instructions. The concentration of RNA in each purified sample was measured by optical density at wavelength 260 nm and adjusted to 0.1 µg/µL in RNAse free H_2_O. 1 µL (0.1 µg) RNA was mixed with 5 µL 25 mM MgCl (Perkin Elmer, Foster City, USA), 8 µL 10 mM dNTP mix (dATP, dTTP, dGTP, dCTP, Pharmacia Biotech/GE Healthcare, Hillerød, Denmark), 1 µL 50 µM 16mer d(T) oligonucleotide primers (DNA Technology, Risskov, Denmark), 2 µL 10 x PCR-buffer (Applied Biosystems, CA, USA), 1 µL 50 U/µL reverse transcriptase (Applied Biosystems, CA, USA), and 1 µL 20 U/µL RNase inhibitor (Applied Biosystems, CA, USA) reaching a total volume of 20 µL. The sample was incubated for 30 min at 42 °C and stored at −20 °C.

Specific primers were used for mouse TC (forward (5′-CTTTGCTGGATCTTCCTTGG-3′), starting at base 1256 in the mouse TCN2 sequence, accession NM 15749 in the NCBI Nucleotide Database; reverse (5′-TCCTGGGGTTTGTAGTCAGC-3′), starting at base 1456 in the mouse TCN2 sequence, accession NM 15749 in the NCBI Nucleotide Database). 1 µl cDNA synthesized as described above was mixed with 5 µL SYBR Green (Light Cycler 480 SYBR Green 1 Master, Roche, Indianapolis, USA), 0.5 µL 5 pmol/µL forward and reverse primers, and RN'ase free H_2_O to a volume of 10 µL. The PCR program was conducted as follows: Preincubation for 10 min at 95 °C followed by 50 cycles of: 10 s at 95 °C, 20 s at 60 °C, and 5 s at 72 °C. Serial dilutions of RNA from the mouse cardiomyocyte cell line HL-1 were used as calibrators in the real time PCR, and the lowest dilution was assigned a concentration of TC-mRNA of 1.22·10^−4^ arbitrary units. The cell line was a generous gift from Dr. William Claycomb, Dept. of Biochemistry & Molecular Biology, School of Medicine, New Orleans, USA.

The PCR products were examined by 1.5% agarose gel electrophoresis and the DNA fragments were shown to have the expected size of 201 bases in accord with the primer design. The DNA bands were purified as previously described [22] and sequenced by Eurofins, MWG Operon to verify the correct identity of the amplified cDNA.

### Data analyses

GraphPad Prism 4 software (GraphPad Software Inc., California, USA) was used for data analyses.

## Results

### DNA/BLAST analysis

We found that BLAST analysis at protein and nucleotide level in mice detected no protein or DNA sequence comparable to the HC/TCN1 seen in man (data not shown). A gene coding for TC (TCN2) has previously been identified in mice [23]. These results sparked the present effort to identify the Cbl-binding proteins in mouse blood and tissues.

### Cobalamin-binding capacity and concentration of total cobalamin

We performed a broad screening of the UB12BC and Cbl content in tissues which in humans contain high levels of Cbl and/or of the Cbl-binding proteins HC or TC.

Mouse lactating mammary glands, kidney, and liver had low Cbl-binding capacities as compared to submaxillary and parotid glands, Table 1. The highest Cbl concentration was observed in the liver followed by the kidney, parotid, submaxillary, and lactating mammary glands. Among all other tissues examined, only the stomach (data not shown) and plasma showed Cbl-binding capacities >1 pmol/g, whereas the esophagus, small intestine, colon, spleen, pancreas, heart, trachea, lung, lymph nodes, skin, muscle, and brain had Cbl-binding capacities <1 pmol/g (data not shown).

**Table 1:** The unsaturated Cbl-binding capacity (UB12BC) and cobalamin content (Cbl) in selected mouse tissues and plasma.

	UB12BC	Cbl
	Median (range) pmol/g	Median (range) pmol/g
**Parotid gl. (n = 6)**	400 (190–510)	190 (130–300)
**Submaxillary gl. (N = 7)**	140 (2–270)	170 (150–220)
**Mammary gl. (n = 6)**	0.5 (0–3)	59 (40–85)
**Liver (n = 7)**	<1	580 (490–670)
**Kidney (n = 7)**	<1	460 (220–570)
**Plasma (n = 7)**	24 (20–29)	29 (28–37)

Results are shown as pmol (median and range) content per g of wet tissue/ml plasma. The number (n) of mice examined are indicated in brackets.

### Characterization of the mouse Cbl-binder

In mice, the tissues most likely to have a high content of HC would be the exocrine glands, since human exocrine secretions contain high HC levels. We therefore characterized the Cbl-binding capacity of the mouse submaxillary glands.

We first compared the binding affinities towards Cbl and the Cbl-analogue Cbi of mouse submaxillary Cbl-binding protein (mouse Cbl-binder) with that of human HC and TC.

**Figure 1:**
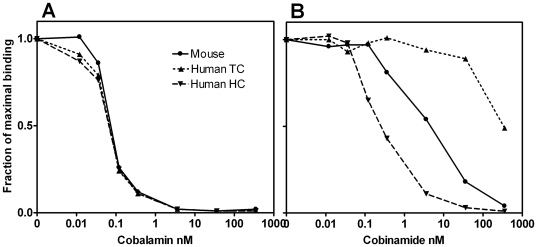
The binding affinities of mouse and human Cbl-binding proteins towards Cbl and Cbi. The binding affinities of the Cbl-binder from mouse submaxillary glands (mouse), human TC, and human HC towards (A) Cbl and (B) Cbi (see Materials and Methods for details).

Mouse Cbl-binder, human HC, and human TC showed comparable binding curves for binding of Cbl, Figure 1A, supporting a comparable affinity towards Cbl for the three proteins. In contrast the binding curves for Cbi showed marked differences, Figure 1B. Kd for binding of Cbi to each of the proteins were estimated as the concentration of Cbi able to displace 50% of labeld Cbl from the binder. In accord with previous work [15] Kd for human HC (<0.5 nmol/L) was low compared to Kd for human TC ( 300 nmol/L). The mouse Cbl-binder bound Cbi better than human TC, but not as efficiently as human HC and showed a Kd of 2 nmol/L.

The glycosylation status of the mouse Cbl-binding protein was examined by precipitation with Con-A. Mouse Cbl-binder and human TC did not bind to the mannose-recognising lectin Con-A, while a large fraction of human HC did bind to this lectin (data not shown). Thus, the initial studies showed that the mouse Cbl-binder has characteristics resembling those of HC (recognition of corrinoids) and those of TC (lack of ability to bind to Con-A).

### Purification and amino acid sequence alignment of the mouse Cbl-binder

To further explore the nature of the mouse Cbl-binder, we purified the Cbl-binder present in submaxillary glands. Mouse submaxillary glands were chosen because of their relatively high concentration of unsaturated Cbl-binding protein and because the submaxillary glands are considerably larger (average 56 mg) than the parotid glands (average 21 mg).

**Figure 2:**
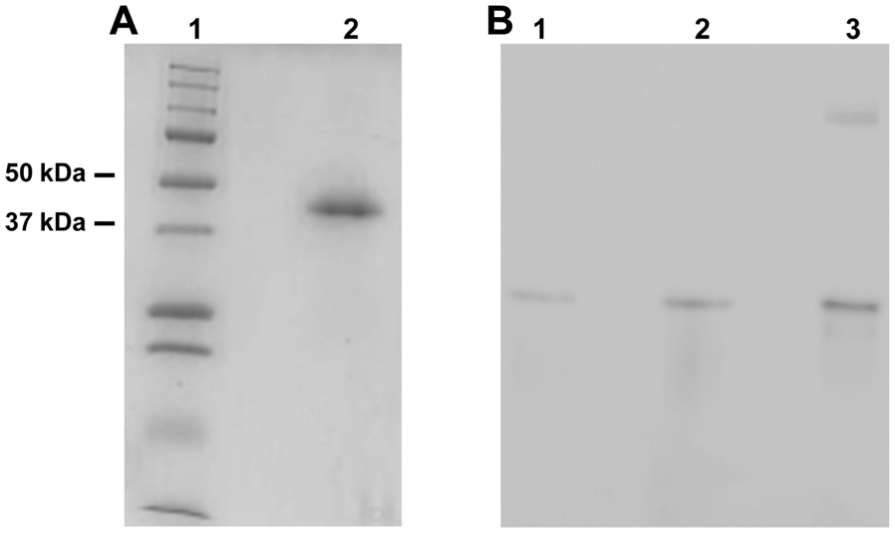
Protein purity and Western blot analyses. (A) 12% reduced SDS-PAGE showing purity of the purified Cbl-binding protein from mouse submaxillary glands. Lane 1) marker; Lane 2) purified mouse TC (see Materials and Methods for details). (B) Western blot employing anti-mouse antibodies on proteins separated by 10% native PAGE. Lane 1) approx. 60 fmol purified mouse TC (approx. 40kDa); Lane 2) extract of the Cbl-binder from mouse submaxillary glands corresponding to a total amount of Cbl+UB12BC of 64 fmol; Lane 3) mouse serum corresponding to a total amount of Cbl+UB12BC of 72 fmol.

The Cbl-binding protein was purified to homogeneity from an extract of 300 glands in a one-step procedure by affinity chromatography employing Cbl coupled to sepharose. A total of approx. 0.3 mg protein was purified with a purification recovery of approx. 85%. As judged from reduced SDS-PAGE, the purified protein had an approx. 40 kDa molecular mass (Figure 2A), which is similar to the molecular mass of human TC.

Sequencing of the purified protein proved that it was mouse TC as the results from two bands covered the amino acid sequence of mouse TC corresponding to 29 % and 20 % respectively, clearly identifying both bands as TC (data not shown).

After identifying the purified mouse Cbl-binder as mouse TC in the database, we aligned the protein sequence of mouse TC to that of the human binders (Supporting Figure S1). The sequence identities are: mouse TC/human TC = 73%; mouse TC/human HC = 28%; and mouse TC/human IF = 28%. For comparison, the sequence identity for human TC/human HC is 32% and for human TC/human IF 26%.

**Figure 3:**
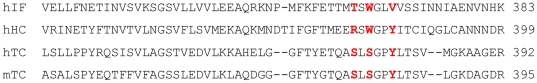
Partial protein sequence alignment of the predicted Cbi-binding region in human proteins. The alignment was done in ClustalW2 using default settings. The region of the three human proteins has previously been predicted to be involved in binding to Cbl and the highlighted residues are the ones expected to be responsible for the binding to Cbl (IF, TC, HC) and other corrinoids (HC only) [24]. For comparison, the similar residue positions in mouse TC are highlighted as well. The numbers in the right margin refer to the specific amino acids of the full-length protein including signal peptides. Complete protein sequence alignment is shown in Supporting Figure S1.

Figure 3 shows a partial protein sequence alignment of the previously predicted Cbl-binding-region of human HC, TC, and IF [24] and the comparable region of mouse TC. The residues are identical in human TC and mouse TC. Of importance to highlight from the global sequence alignment is that the Cobalt-coordinating histidine-residue in human TC is present in mouse TC (His74).

Taken together, the results from the sequence alignments including DNA, mRNA and protein analysis all imply that the mouse Cbl-binder is a TC.

### Characterization of mouse Cbl-binding proteins by use of specific antibodies

Polyclonal anti-mouse TC was produced in rabbit and proved to recognize TC in the purified Cbl-binder from submaxillary glands in a crude extract of submaxillary glands and in mouse serum, see Figure 2B. The antibody did not show cross-reactivity with equimolar amounts of human TC or HC (results not shown).

Anti-mouse TC was insolubilized on protein-A agarose. As expected, the insoluble antibody was capable of precipitating the Cbl-binding capacity and Cbl in extracts of mouse submaxillary glands (>98% UB12BC and >93% Cbl precipitated) (data not shown). Interestingly, the antibody also precipitated the Cbl-binding capacity and Cbl in mice sera (>97% UB12BC and >98% Cbl precipitated) (data not shown).

### Immunohistochemical localisation of TC

**Figure 4:**
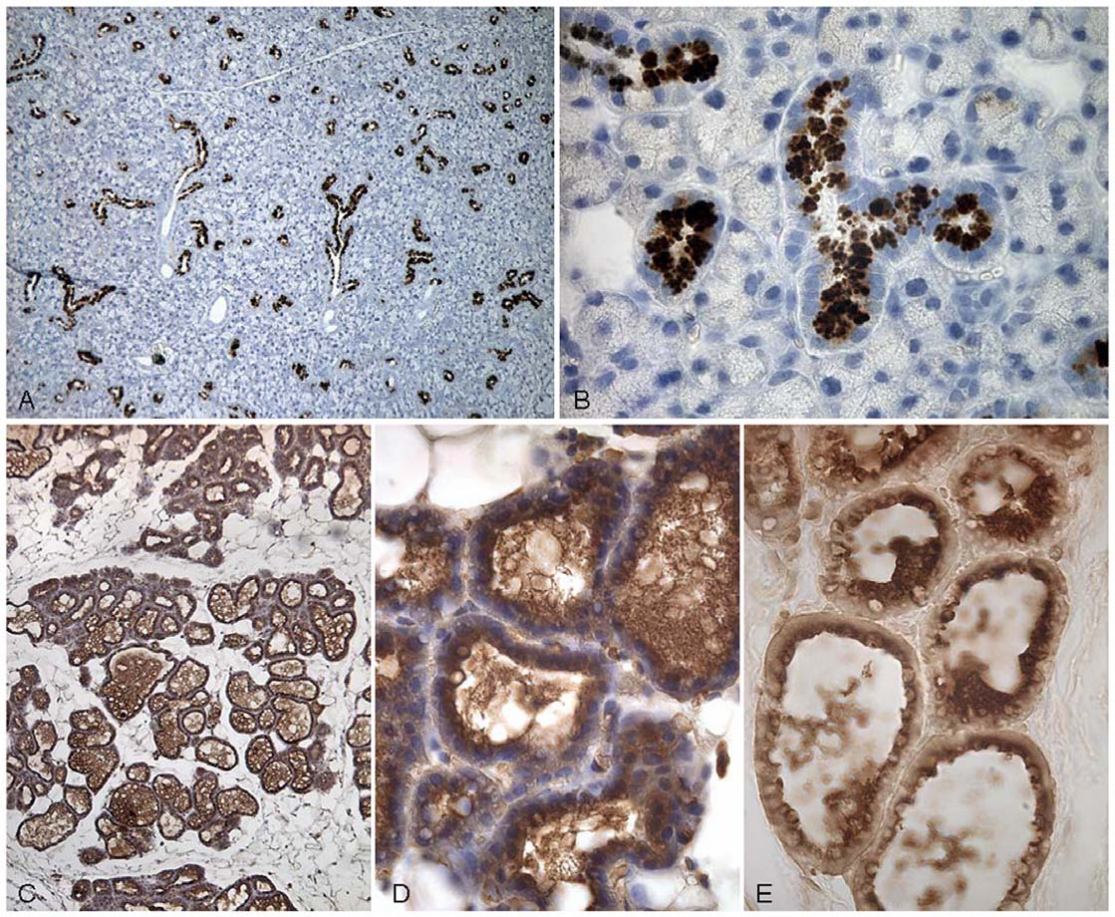
Immunohistochemical localization of TC in mouse tissues using anti-mouse TC. (A, B) The submaxillary gland shows positive staining in the granules of the granular, convoluted tubule cells. (C, D, E) Lactating mammary gland with some reaction in the luminal secretion. Magnifications: (A, C) X 75, (B) X 300, (D, E) X 475.

The TC expression in mice was examined by immunohistochemistry, Figure 4. TC was found in the granules of the granular, convoluted tubule cells in the submaxillary glands and in the luminal secretion in the lactating mammary glands.

**Figure 5:**
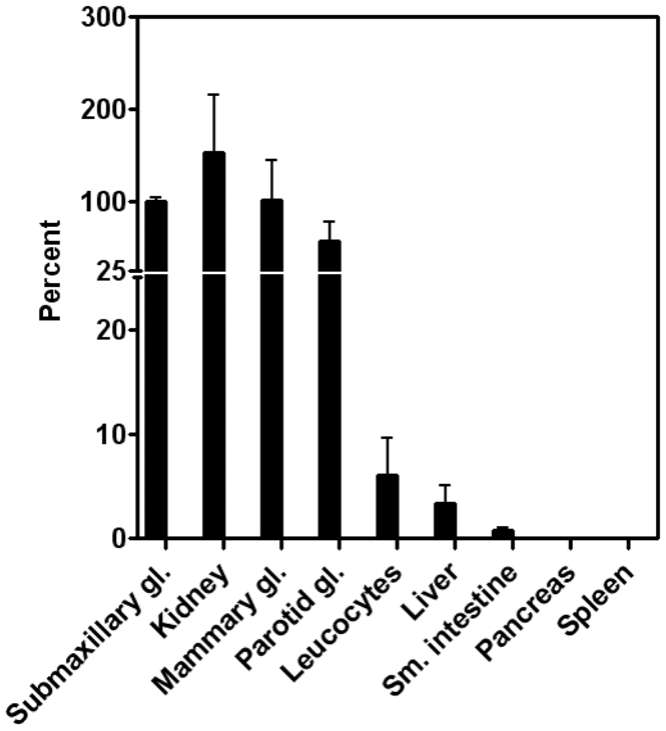
Comparison of the transcription levels of TC in various mouse tissues. The transcription level of TC is shown for each tissue (mean and SEM) relative to the transcription in submaxillary glands (arbitrary value of 100). N = 3 (submaxillary gland, kidney, leucocytes, small intestine; 4 (mammary gland, parotid gland, pancreas, spleen); 2 (liver).

### Transcription level of mouse TC

We explored the transcription of TC in tissues known to produce either TC or HC in humans.

The transcription level of TC in different tissues is depicted in Figure 5 relative to their submaxillary gland transcription. A high transcription level of mouse TC was seen in tissues known to have high transcription levels of either TC or HC in humans [2], notably the mammary, submaxillary, and parotid glands. The small intestine, pancreas, and spleen showed low transcription levels.

## Discussion

We report that no HC-like protein is present in mice as determined by BLAST analysis of the mouse genome. Instead, the unsaturated Cbl-binding protein purified from mouse submaxillary glands proved to be TC and not HC, as expected; and serum contained TC as the only Cbl-binding protein. Mouse TC shows features comparable to both human TC (sequence, size, lack of ability to bind to Con-A) and human HC (affinity towards Cbi, present at high levels in exocrine glands).

Three soluble Cbl-binding proteins have been described. Intrinsic factor is present in the gastrointestinal tract and promotes the intestinal uptake of Cbl, while TC ensures transport of Cbl in the blood stream and to all cells [25]. The function of the third soluble transporter of Cbl, HC, remains to be clarified. In humans, HC carries the major part of circulating Cbl and it is present also in exocrine secretions like milk and saliva [2]. While human IF and TC recognize only Cbl, HC is able to recognize a wide spectrum of corrinoids including Cbi [26]. IF, TC, and HC are structurally related and must have emerged from a common ancestral gene. TC is considered to be the oldest of the proteins with IF as the next in line. HC is likely to have developed from a duplication of the IF gene and in humans the genes coding for IF and HC both reside on chromosome 11[27].

The occurrence in mammals of each of the circulating proteins TC and HC has been documented by both direct and indirect measures [4]-[9]. Notably TC has been purified from a number of species including man, rabbit, cow, and now also from mice. Likewise, HC has been purified from man, hog, and rabbit. Most of these studies were based on indirect methods to classify the nature of a given soluble Cbl-binding protein and, in doing so, a Cbl-binding protein capable of binding Cbi has been classified as a HC-type protein. Our results show that this is not enough to prove protein identity. We demonstrate that mouse TC is able to recognize Cbi; our results thereby warrant caution as to the interpretation of previous data on the occurrence of HC in mammals. Scrutiny of previous data on Cbl-binding proteins in the rat reveals that the claimed presence of HC rests on evidence that is somewhat indirect. The Cbl-binder in rat saliva was classified as HC on the basis of its ability to bind Cbi, despite other characteristics according to which the protein resembled TC, e.g. a low carbohydrate content [8]. To explore a possible existence of HC in rats at protein or genomic level (TCN1), we performed a BLAST analysis; but as for the mouse, we identified neither the protein nor the gene (data not shown). We thus find it likely that neither the mouse nor the rat harbors a gene coding for HC.

Our inability to find a gene coding for HC in the mouse genome at the start of the present study made us believe that we would find a new Cbl-binding protein to replace HC in mice. Instead, we observed TC in all of the sites where HC was expected to occur. Mouse TC-mRNA was transcribed at high levels in salivary and mammary glands, whereas HC is located at these sites in humans. Likewise, mouse TC was the only Cbl-binding protein present in submaxillary glands and in the circulation, while saliva and serum contain HC in humans.

Together with previous reports, our data suggest that the nature of the Cbl-binding protein in exocrine secretions may differ amongst species. A known example is the difference between cow and man who both have HC and TC. TC is the dominating Cbl-binding protein in cow's milk [28], while HC is found in human milk. Our data show that TC may substitute HC, especially in lower animals that may only have TC and not HC.

The binding of TC to other corrinoids besides Cbl is a novel discovery. This ability has been related to HC in other mammals. Wuerges et al. suggested that three residues in human HC are responsible for its ability to bind Cbi: R^380^, W^382^, and Y^385^; all highlighted in Figure 3. In human IF and human TC, the position of the HC residue R^380^ is different (T^385^ and S^374^, respectively), whereas residue W^382^ in HC is also W in IF (W^376^), but serine (S^387^) in TC. The position of Y^385^ in human HC is also Y in human TC (Y^390^), but it is valine (V^379^) in human IF. At these three positions, mouse TC has residues identical to those of human TC. These suggested residues are therefore not exclusively responsible for the binding to Cbi, at least not in the mouse TC protein. Point mutations in recombinant proteins and/or crystallography of protein-Cbi complexes may show which residues are truly involved in the binding of Cbl derivatives.

In conclusion, we report that in the mouse, TC is the only Cbl-binding protein in serum and in tissues known to express HC. Mouse TC behaves like an intermediate between human TC and HC as it binds Cbi, but not Con-A.

## Supporting Information

Figure S1 **Complete sequence alignment depicting the relation between various mammalian Cbl-binding proteins.** Protein sequence alignment of mouse TC, rat TC, human TC, human IF, and human HC. The residues highlighted with yellow show the Cobalt-coordinating histidine-residues in mouse TC, rat, TC, and human TC. The residues highlighted with red are expected to be responsible for the binding to Cbl (IF, TC, HC) and other corrinoids (HC only) [24]. The numbers in the right margin refer to the specific amino acids of the full-length protein including signal peptides.
(DOCX)
