# Supplementary material for: Mouse Transcobalamin Has Features Resembling both Human Transcobalamin and Haptocorrin
Source: PLoS One. 2011 May 31;6(5):e20638. doi: 10.1371/journal.pone.0020638 (PMC3105116; doi:10.1371/journal.pone.0020638)
Supplement: Figure S1 — Complete sequence alignment depicting the relation between various mammalian Cbl-binding proteins. Protein sequence alignment of mouse TC, rat TC, human TC, human IF, and human HC. The residues highlighted with yellow show the Cobalt-coordinating histidine-residues in mouse TC, rat, TC, and human TC. The residues highlighted with red are expected to be responsible for the binding to Cbl (IF, TC, HC) and other corrinoids (HC only) [24]. The numbers in the right margin refer to the specific amino acids of the full-length protein including signal peptides. (DOCX) [file pone.0020638.s001.docx]

mTC -----MELLKALLLLSGVFGALAEFCVIPRIDSQLVEKLGQRLLPWMDRLSSEQLNPSVF 55

rTC -----MELLKALLLLSGVLGALAEFCVIPKMDGQLVEKLGQRLLPWMDRLSSEQLNPSIY 55

hTC -----MRHLGAFLFLLGVLGALTEMCEIPEMDSHLVEKLGQHLLPWMDRLSLEHLNPSIY 55

hIF MAWFALYLLSLLWATAGTSTQTQSSCSVPSAQEPLVN--GIQVLMENSVTSSAYPNPSIL 58

hHC MRQSHQLPLVGLLLFSFIPSQLCEICEVSEENYIRLKPLLNTMIQSNYNRGTSAVN--VV 58

* : . * :. : :: :: . * :

mTC VGLRLSSMQAGTKEDLYLHSLKIHYQQCLLRSTSSDDNSSCQPKLSGGSLALYLLALRAN 115

rTC VGLRLSSMQAGTKENLYLHNLKLHYQQCLLRSTSSDDNSGCQTKISGGSLALYLLALRAN 115

hTC VGLRLSSLQAGTKEDLYLHSLKLGYQQCLLGSAFSEDDGDCQGKPSMGQLALYLLALRAN 115

hIF IAMNLAGAYN------------LKAQKLLTYQLMSSDNN----DLTIGQLGLTIMALTSS 102

hHC LSLKLVGIQIQT--------LMQKMIQQIKYNVKSRLSDVSSGELALIILALGVCRNAEE 110

:.:.* . : : . * .. . : *.* : .

mTC CEFFGSRKGDRLISQLKWFLEDEKKAIGHNHEGHPNTNYYQYGLSILALCVHQKRLHDSV 175

rTC CELLGSRKGDRMVSQLKWFLEDEKKAIGHHHEGHPHTSYYQYGLSILALCVHRKRVHDSV 175

hTC CEFVRGHKGDRLVSQLKWFLEDEKRAIGHDHKGHPHTSYYQYGLGILALCLHQKRVHDSV 175

hIF CRDPGDK-----VSILQRQMEN----WAPSSPNAEASAFYGPSLAILALCQKNSEATLPI 153

hHC NLIYDYHLIDKLENKFQAEIEN-----MEAHNGTPLTNYYQLSLDVLALCLFNGNYSTAE 165

: . :: :*: . : :* .* :**** . . .

mTC VGKLLYAVEHDYFTYQGHVSVDTEAMAGLALTCLE------RFNFNSDLRPRITMAIETV 229

rTC VGKLLYAVEHDYFTYQGHLSVDTEAMAGLAFTCLE------RFNFNSDLRPRITTAIETV 229

hTC VDKLLYAVEP---FHQGHHSVDTAAMAGLAFTCLK------RSNFNPGRRQRITMAIRTV 226

hIF AVRFAKTLLAN----SSPFNVDTGAMATLALTCMYN---KIPVGSEEGYRSLFGQVLKDI 206

hHC VVNHFTPENKN-YYFGSQFSVDTGAMAVLALTCVKKSLINGQIKADEGSLKNISIYTKSL 224

. . . . .*** *** **:**: : . : . :

mTC REKILKSQAPEGYFGNIYSTPLALQMLMTSPASGVGLGTACIKAGTSLLLSLQDGAFQNP 289

rTC REKILKAQAPEGYFGNIYSTPLALQMLMTSP--GVGLGPACLKARKSLLLSLQDGAFQNP 287

hTC REEILKAQTPEGHFGNVYSTPLALQFLMTSPMRGAELGTACLKARVALLASLQDGAFQNA 286

hIF VEKISMKIKDNGIIGDIYSTGLAMQALSVTPEPSKKE-WNCKKTTDMILNEIKQGKFHNP 265

hHC VEKILSEKKENGLIGNTFSTGEAMQALFVSSDYYNENDWNCQQTLNTVLTEISQGAFSNP 284

*:* :* :*: :** *:* * .:. * :: :* .:.:* * *.

mTC LMISQLLPILNHKTYLDLIFP-DCQASRVMLVPAVEDP---------VHISEVISVTLKV 339

rTC MMISQLLPVLNHKTYLNLISP-DCQAPRVMLVPATEDP---------VHLSEVS-VTLKV 336

hTC LMISQLLPVLNHKTYIDLIFP-DCLAPRVMLEPAAETI---------PQTQEIISVTLQV 336

hIF MSIAQILPSLKGKTYLDVPQV-TCSPDHEVQPTLPSNPGPGPTSASNITVIYTINNQLRG 324

hHC NAAAQVLPALMGKTFLDINKDSSCVSASGNFNISADEP-----ITVTPPDSQSYISVNYS 339

:*:** * **:::: * . .

mTC ASALSPYEQTFFVFAGSSLEDVLKLAQDGG--GFTYGTQASLSGPYLTSV--LGKDAGDR 395

rTC SSVLPPYERTVSVFAGASLEDVLNRARDLG--EFTYGTQASLSGPYLTSV--LGKEAGDR 392

hTC LSLLPPYRQSISVLAGSTVEDVLKKAHELG--GFTYETQASLSGPYLTSV--MGKAAGER 392

hIF VELLFNETINVSVKSGSVLLVVLEEAQRKNP-MFKFETTMTSWGLVVSSINNIAENVNHK 383

hHC VRINETYFTNVTVLNGSVFLSVMEKAQKMNDTIFGFTMEERSWGPYITCIQGLCANNNDR 399

.. * *: . *:: *: . * : * ::.: : ..:

mTC EYWQLLRAPDTPLLQGIADYKPQDGETIELRLVRW 430

rTC EYWQLLRVPDTPLLQGIADYKPKNGETIELRLVKM 427

hTC EFWQLLRDPNTPLLQGIADYRPKDGETIELRLVSW 427

hIF TYWQFLSG-VTPLNEGVADYIPFNHEHITANFTQY 417

hHC TYWELLSG-GEPLSQGAGSYVVRNGENLEVRWSKY 433

:*::* ** :* ..* : * : .
